# Evaluating Localization Accuracy of Automated Driving Systems

**DOI:** 10.3390/s21175855

**Published:** 2021-08-30

**Authors:** Karl Rehrl, Simon Gröchenig

**Affiliations:** Mobility & Transport Analytics Group, Salzburg Research Forschungsgesellschaft mbH, 5020 Salzburg, Austria; simon.groechenig@salzburgresearch.at

**Keywords:** automated driving, localization accuracy evaluation, ground-truth

## Abstract

Automated driving systems are in need of accurate localization, i.e., achieving accuracies below 0.1 m at confidence levels above 95%. Although during the last decade numerous localization techniques have been proposed, a common methodology to validate their accuracies in relation to a ground-truth dataset is missing so far. This work aims at closing this gap by evaluating four different methods for validating localization accuracies of a vehicle’s position trajectory to different ground truths: (1) a static driving-path, (2) the lane-centerline of a high-definition (HD) map with validated accuracy, (3) localized vehicle body overlaps of the lane-boundaries of a HD map, and (4) longitudinal accuracy at stop points. The methods are evaluated using two localization test datasets, one acquired by an automated vehicle following a static driving path, being additionally equipped with roof-mounted localization systems, and a second dataset acquired from manually-driven connected vehicles. Results show the broad applicability of the approach for evaluating localization accuracy and reveal the pros and cons of the different methods and ground truths. Results also show the feasibility of achieving localization accuracies below 0.1 m at confidence levels up to 99.9% for high-quality localization systems, while at the same time demonstrate that such accuracies are still challenging to achieve.

## 1. Introduction

An automated driving system (ADS) supporting automation levels 3 to 5 according to SAE International Standard J3016^TM^ [1] is supposed to be able to automatically execute driving maneuvers in specific operational design domains (ODDs) [2] with decreasing human intervention. An ADS being classified as SAE J3016^TM^ level 4 (high automation), for example, requires that the driving system is able to precisely and safely execute driving maneuvers such as lane changes or turns at intersections. In order to execute such driving maneuvers, localization accuracies of 0.1 m at 95% confidence are a crucial requirement of an automated vehicle (AV) [3]. Due to complex and dynamically changing driving environments, achieving such accuracies reliably over time or distance is considered as one of the main challenges of AVs.

During the last decade, various localization techniques for AVs have been proposed [4,5,6,7,8]. A comprehensive review of the current state-of-the-art of the three most common localization approaches ((1) GNSS-IMU fusion, (2) Simultaneous Localization and Mapping (SLAM), and (3) a priory map-based localization and their potential for automated driving) can be found in [4]. All localization techniques have in common that their capability of being used for the localization of AVs has to be proven with a validated ground-truth dataset. According to [3], the performance of safety-critical localization systems can be evaluated using the key performance indicators (KPIs) accuracy, integrity, and availability. The authors propose a three-dimensional geometrical bounding box around a vehicle as protection level, i.e., in order to be safe for the localization of the AV, the output of the localization system has to stay within this bounding box. The three KPIs are used to indicate to which extent a localization system is able to fulfill the localization requirements of AVs. The KPI availability is defined as the ratio to which the localization system is able to keep the localization error within the alert limits (bounding box). If the localization error exceeds the alert limits, no safe operation of the AV is possible and the localization system’s status is set to unavailable. KPI integrity describes the probability to which the localization system is capable to keep the localization error within the alert limits over distance or time and accuracy describes the nominal performance of the localization system and is typically described with a confidence level, e.g., 0.1 m accuracy at 95% confidence. Consequently, the KPI accuracy describes the measured performance of the localization system while integrity describes to which degree the defined safety limits can be met.

In their work, the authors further derive accuracy and integrity requirements for different vehicle types and ODDs. For passenger cars on local US roads, the authors propose lateral and longitudinal localization accuracies of 0.1 m at 95% confidence as well as alert limits of 0.29 m. As integrity levels they define 10^−9^ per mile or 10^−8^ per hour. The 5G PPP (5G Infrastructure Public Private Partnership, a joint initiative between the European Commission and the European ICT industry) [9] proposes that AVs should achieve a localization accuracy below 0.3 m (originally, this threshold has been proposed by [10]). The 5G PPP argues that safety functions of AVs should achieve an integrity level of 10^−5^ (99.999%) which is considerably lower compared to the integrity levels defined by [3]. Another definition for accuracy requirements of safety-critical automated driving comes from the European GSA with <0.2 m accuracy and 99.9% availability [11]. After reviewing different localization requirements for AVs, it is obvious that currently there exists no commonsense on the accuracy, integrity, and availability requirements for automated driving systems, which has recently also be confirmed by [12]. Due to the most reasonable scientific grounding and the stringent accuracy requirements, for the current work, we rely on the requirement definitions by [3] with 0.1 m accuracy at 95% confidence.

Concerning evaluation methods for localization performance, two different categories of approaches have been proposed by other researchers. The first category evaluates the performance of a localization system in relation to a high-quality reference position trajectory [7,13,14]. This approach allows for an efficient comparison of longitudinal and lateral position errors but suffers from the drawback that a position trajectory, if estimated by a localization system, may hardly be considered as ground-truth. Even if a high-quality localization system is used, it is still a dynamic system and localization errors may occur at any time. Therefore, the second category uses a static, map-based ground-truth, either a pre-defined driving path [15] or a high-definition (HD) map [16,17]. Even if the static ground-truth is of high quality, a certain error compared to the ground truth may be expected. The International Maritime Organization (IMO) distinguishes between the navigation system error (caused by the navigation system) and the chart error (caused by surveying inaccuracies or errors in the geodetic reference system) [18]. Typically, the chart error of a HD map for highly automated driving may account for an error up to 0.1 m. Therefore, when using a map-based ground truth, the chart error may play a significant role for the error estimation and has to be considered as well. However, it is challenging to estimate the map error and to deal with varying accuracies of different map sections and elements. Moreover, even in case of a ground-truth with known accuracies, the question of how location measurements should be validated against this ground-truth remains unaddressed. Sharath et al. [17], for example, argue that most map-matching algorithms match trajectories only to the middle axis of the road and not in relation to the driving lane. In [16], the authors apply a lane-level map matching algorithm to evaluate the uncertainty of GNSS localization and in [19], the authors apply a lane-level HD map matching algorithm to determine availability, accuracy and integrity. However, while lane-level map matching is indeed able to match position trajectories to the correct driving lane, since an AV’s actual driving path may deviate from the centerline, the question remains unaddressed how sub-decimeter localization errors with respect to the driving path or the HD map can be evaluated. From related work, we conclude that a common methodology for evaluating localization performance of AVs in real-world driving scenarios with respect to a validated ground truth dataset is missing so far. The current work aims at closing this gap.

The approach proposed in this paper considers the localization system of an AV as black box and evaluates localization accuracy of the resulting position trajectory in relation to two different ground-truth datasets, a pre-defined driving path and a lane-level HD map. Given these two ground truths, the AV’s localization performance is evaluated by calculating Euclidean error distances between the localization measurements and (1) the AV’s driving path (lateral error), (2) the centerline of the driving lane (lateral error), (3) the lane boundaries being overlapped by the AV’s geometrical body (overlapping distance error) and (4) AV’s longitudinal offset at known stop points (longitudinal error). By applying these methods, the following accuracy measures can be derived: (1) path accuracy (lateral distance error with respect to a pre-defined driving path); (2) lane-centerline accuracy (lateral distance error with respect to the centerline of the driving lane); (3) lane-boundary overlaps (driving distance or percentage of AV’s localized body overlapping lane boundaries); and (4) longitudinal accuracy (longitudinal distance error at pre-defined stop points). For evaluating accuracies, statistical measures such as the mean Euclidean distance (MED), standard deviation and confidence levels are compared using two different calculation methods: (1) measurement-based evaluation (accuracies are calculated based on Euclidean error distances of position measurements) and (2) distance-based evaluation (accuracies are weighted by the distance travelled by the AV; see Section 2.2).

The proposed methods and measures are evaluated using two localization datasets. The first dataset has been acquired from test drives with an ADS (EZ10-Gen3 self-driving shuttle from EasyMile (Toulouse 31000, France) (https://easymile.com/vehicle-solutions/ez10-passenger-shuttle, accessed on 5 July 2021)), which is capable of autonomously following a pre-defined driving path being used as ground truth. To compare the localization performance of ADS to other localization systems, the EZ10-Gen3 shuttle has been equipped with further localization systems, namely a Multi-GNSS/RTK system, an IMU-supported multi GNSS-smartphone with L1/L2 GNSS localization capability, and a standard Multi-GNSS-based system. The second dataset has been acquired from ETSI ITS-G5 CAM messages [20], received from manually driven, C-ITS (Cooperative Intelligent Transport Systems)-enabled vehicles [21] via five roadside ITS stations. Although these vehicles are not automated, the second test dataset is used to demonstrate the wider applicability of the approach and answers the question whether C-ITS enabled vehicles on the market cope with the localization requirements of C-ITS use cases such as Intersection Collision Risk Warning (ICRW) [22,23]. Results show that the proposed evaluation methods are able to estimate localization accuracy of arbitrary localization systems including those of AVs as well as manually driven connected vehicles. While a high-quality localization system of an AV using sensor fusion is capable of achieving an accuracy below 0.1 m at confidence levels up to 99.9%, Multi-GNSS/RTK-based systems are still capable to achieve lane-level accuracies below 0.5 m at the same confidence level. Consumer-grade systems as well as C-ITS-enabled vehicles are currently not able to cope with the requirements of lane-level accuracy.

## 2. Materials and Methods

### 2.1. Ground-Truth

In order to evaluate localization accuracy of moving objects, beside the localization data, a ground-truth dataset is needed. Although in previous works, localization systems under test are often evaluated in relation to a high-quality reference localization system, a localization system may be hardly considered as ground truth. For this work, ground-truth is defined as a pre-defined, static dataset with validated accuracy representing either an AV’s driving path or the AV’s driving lane from an HD map.

One possible ground truth dataset is the known driving path of an AV. This driving path can be either static (as used by the EZ10-Gen3 for path following) or dynamic (being calculated by the AV based on a HD road map or environmental perception). A static driving path is considered best suited as ground truth since it represents the path that has to be followed by the AV at best effort. For example, the static driving path of the EZ10-Gen3 is acquired by manual drives with the internal localization system. After acquisition, it has to be manually edited until it represents the intended driving path of the vehicle on the road (validated by test drives). However, it has to be considered that the resulting AV’s location measurements may contain an actuation error being a result of the AV’s actuation strategy, but this is the case with location measurements of any AV and cannot be considered separately if the AV is treated as a black box. Nevertheless, localization inaccuracies will most likely lead to deviations from the driving path since the localization measurements are the main data source for the actuation system. A dynamic driving path, if known, can be used as ground truth as well. However, the dynamic driving path has to be calculated based on an accurate ground-truth dataset such as an HD map with proven accuracy. Moreover, since it may continuously be recalculated and differs for each test drive, a static driving path should be preferred as ground-truth.

A second possible ground-truth dataset is an HD map representing the road infrastructure with a proven accuracy at centimeter-level. Such an HD map can be composed from sensor data being collected by a HD mapping vehicle [24], after extraction of the relevant road features from the LiDAR (Light Detection and Ranging) point cloud and modelling the map features in a HD map format such as Lanelet2 [25]. Lanelet2 HD maps represent the road infrastructure as geographically referenced areas. Driving lanes are modelled as lanelets (directed lane sections of variable length with left and right boundaries). For modelling the ideal driving path of an autonomous vehicle on this lane, the centerline of each lanelet can be calculated as middle axis from the left and right boundaries. Similar to the static or dynamic driving path, the Euclidean distance of each AV’s location measurement to this centerline can be calculated. However, since it may not be assumed that the AV actually follows the centerline during its drive, the resulting error distances between localization measurements and the centerline can only answer the question whether the vehicle stays within the driving lane. If the exact driving path is not known, lane-level accuracy is considered as best measure for determining localization accuracy.

### 2.2. Localization Accuracy Measures

**Figure 1:**
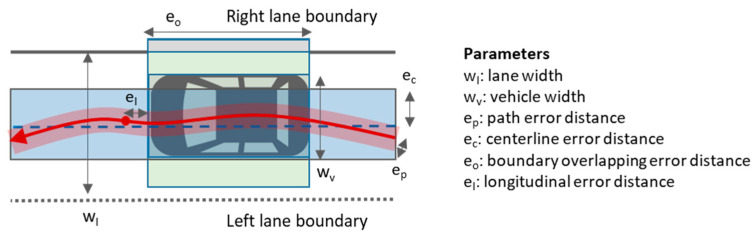
Schema of a driving lane illustrating the relevant variables (lane width and vehicle width) and the four error distances (path error distance, centerline error distance, boundary overlapping error distance and longitudinal error distance).

As stated earlier, the goal of the proposed approach is to consider the localization system of an AV as black box. The data source under evaluation is the resulting position trajectory from an arbitrary drive, i.e., a time-ordered sequence of vehicle localizations. For the localization accuracy evaluation, each localization measurement is set in relation to one of the two ground truths, either the static driving path or the driving lane of the HD map. Given a vehicle movement in three degrees of freedom (3 DoF), with respect to the ground truth, four different error measures can be calculated. Figure 1 illustrates a driving lane and an AV’s geometrical body with the relevant variables (lane width and vehicle width) and the four different error measures:

- **Path error distance (e_p_)** (red area): It measures how far the localization measurements deviate from the actual driving path (red arrowed line). The localization accuracy is derived by calculating the lateral Euclidean error distance for each localization measurement (AV’s reference point) in relation to the driving path.
- **Centerline error distance (e_c_)** (blue area): It is a combination of the driving error (the deviation of the vehicle from the centerline during a drive) and the localization error (the deviation of localization measurements from the driving path). Although it is not possible to separate the individual errors, the combined Euclidean error distance between the AV’s reference point and the lane centerline must not exceed the centerline error threshold so that the vehicle’s localized body is not overlapping the lane boundaries during drive (this assumption only holds if the driving lane is known and the vehicle does not leave or change the driving lane while under evaluation).
- **Boundary overlapping error distance (e_o_)** (grey area): Besides calculating the Euclidean error distances between the AV’s location reference point and the lane centerline, the lane-keeping can also be evaluated by intersecting the AV’s located and oriented geometrical body in three degrees of freedom (3 DoF) for each position measurement with the lane boundaries. The vehicle’s geometrical body should not overlap the lane boundaries, otherwise the longitudinal distance of the boundary overlap is considered as boundary overlapping error distance. In comparison to the point- based path and centerline error distance measures, a semantically richer accuracy evaluation is possible (i.e., it allows to determine at which locations and to which side overlaps occur or the localized vehicle is overlapping soft or hard lane boundaries).
- **Longitudinal error distance (e_l_)**: The Euclidean longitudinal error distance can only be calculated in case of known stops points since otherwise the ground-truth is missing. In case of stop points, the error distance between the AV’s reference point and the stop point is calculated.

Calculating Euclidean error distances as well as confidence levels can be accomplished in three different ways: (1) measurement-based, (2) time-based, or (3) distance based. For determining the reliability of the localization system, it is relevant to determine confidence levels, i.e., the ability of the localization system to keep a certain accuracy over measurements, time, or distance. Confidence levels can be derived from the corresponding cumulative frequency distributions (CFD) of error distances (weighted by time or distance), i.e., how many of the localization measurements stay below a certain error threshold in relation to all measurements or total distance. The following methods for calculating the statistical values may be applied:

- **Measurement-based error distances**: Euclidean error distances are calculated for each position measurement of the trajectory.
- **Time-based error distances**: Euclidean error distances are also calculated for each position measurement of the trajectory but are weighted by time. If localization intervals are stable, there is no difference to measurement-based error distances. Only in case of variable localization intervals, variances in the calculated values may occur.
- **Distance-based error distances**: Euclidean error distances are again calculated for each position but weighted by the travelled distance in relation to the total distance. Consequently, distance errors at high speeds count more compared to distance errors at low speed. This measure could be more appropriate since many safety-related key performance indicators (KPIs) of AVs are measured in relation to the driven distance (e.g., disengagements of autonomous mode or accidents) [26].

When applying the previously introduced accuracy measures to the SAE J3016^TM^ automation levels, it can be recognized that not all measures can be applied to all automation levels. Path accuracy and longitudinal accuracy can only be measured for partly or highly automated vehicles from SAE J3016^TM^ level 3 onwards since vehicles at SAE J3016^TM^ levels 0 to 2 do not have the capabilities to follow a driving-path or to stop automatically at a stop point. However, for vehicles at SAE J3016^TM^ levels 0-2, the lane- accuracy of their localization systems can still be evaluated (since also a human driver or a driving assistance system has to stay within the lane boundaries). Therefore, position measurements of a high-quality localization system have to stay within the lane as well. Table 1 gives an overview of the different evaluation options with respect to the SAE J3016^TM^ automation levels.

### 2.3. Evaluation Methods

This section introduces the calculation methods of the measurement-based and distance-based path and centerline lateral error distances, the boundary overlapping error distances, and the longitudinal error distances.

#### 2.3.1. Calculation of Measurement-Based Path and Centerline Lateral Error Distances

The measurement-based lateral path error distances are calculated as follows: for each WGS84-coordinate pair of a position measurement, the minimal Andoyer distance to the driving path or lane-centerline is determined by using a map-matching algorithm as the one proposed in [27]. Matching each position to the path or lane-centerline results in a positive (towards left boundary in driving direction) or negative (towards right boundary) error distance. As in [16,19], map-matching has been selected as optimal strategy to select the nearest path or lane-centerline segment while obeying to traffic regulations. Therefore, the matched path or lane-centerline segments have: (1) the lowest Euclidean distance between the position measurements and the referenced segments and (2) the route and path distance difference is minimized. In order to retrieve feasible map-matching results for route parts with close path options (e.g., planned U-turns of the driving path), the maximum matching radius has to be limited. For this work, the maximum matching radius has been set to 5 m, resulting in lateral error distances not higher than this threshold. While for high-quality localization systems this threshold will never be exceeded, low-quality systems may exceed it. In this case, the measurements with lateral error distances higher than 5 m cannot be map-matched and are therefore excluded from the accuracy evaluation.

#### 2.3.2. Calculation of Distance-Based Path and Centerline Lateral Error Distances

In contrast to the calculation of measurement-based lateral error distances, for the distance-based calculation the individual error distances of each localization measurement in relation to the path or lane-centerline are weighted according to the driving distance between two measurements in relation to the total driving distance. For example, if the driving distance to the last position measurement is short, the weight is low and increases with the distance. Consequently, measurement error at slow speed or at stops have a lower or no impact (in case of stops) because less driving distance is covered. For example, measurement errors during a 30 s stop will result in 30 measurement-based lateral distance errors (assuming 1 Hz sampling rate) or in 30 distance-based errors with low weight since no or only a low driving distance is covered. The longitudinal driving distance can be calculated in two ways: (i) deriving the distance from measurements or (ii) deriving the distance from the driving path or lane-centerline. The later one is used for this evaluation since it avoids an increase of the distance due to erroneous localization measurements.

#### 2.3.3. Calculation of Boundary Overlapping Error Distances

**Figure 2:**
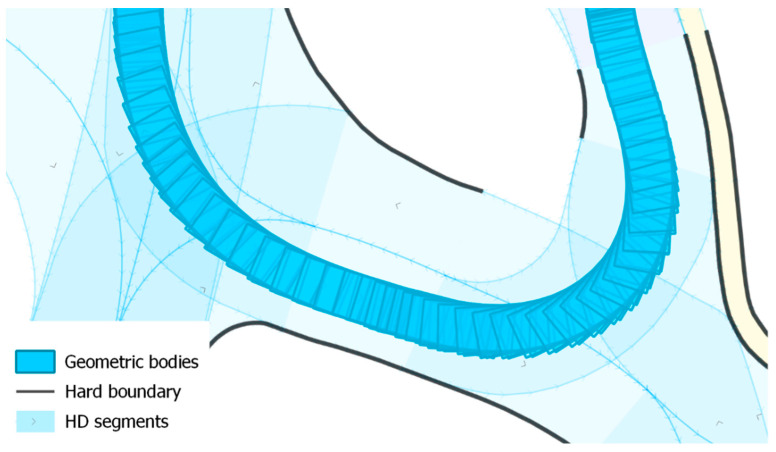
Excerpt of a position trajectory showing the calculated AV’s oriented geometrical bodies (blue polygons), lanes from the HD map (light blue) and hard boundaries (black lines).

Besides calculating the lateral error distances between localization measurements and the path or lane-centerline, this approach intersects the AV’s geographically oriented geometric body (3 DoF) with the geometries of the driving lane and calculates left and right boundary overlaps separately. The orientation of the AV’s geometrical body is calculated from the GNSS data for each position using the heading to the next position. To enable a correct polygon creation, the coordinates are transformed to the Cartesian coordinate reference system ‘MGI/Austria GK M31’. See Figure 2 for an excerpt of a position trajectory where the oriented geometric body of the AV is visualized for each track point. In case of a boundary overlap, the driving distance from the last position measurement is summed up as left and/or right boundary overlapping error distance. At the end of each drive, the sum is put in relation to the whole driving distance which results in the percentage of boundary overlaps. Since a Lanelet2 HD map contains hard (may not be crossed) and soft (may be crossed) lane boundaries, both types of boundary overlapping errors are calculated separately. In contrast to the lane-centerline based evaluation method, this method results in semantically richer information with respect to the actual driving environment.

#### 2.3.4. Calculation of Longitudinal Error Distances

The longitudinal error distance can only be calculated at defined stop locations from the driving path or the HD map. These stop locations can be considered as the ground truth and the longitudinal error distance of stops from the position trajectories in relation to these ground-truth stop locations can be calculated. For instance, if the driving path yields a vehicle stop at 50 m and the trajectory includes a stop after 50.4 m, this leads to a longitudinal error of 0.4 m. All stop locations in the driving path as well as in the trajectories refer to the AV’s geometrical center (reference position). Determining longitudinal errors from stop lines in the HD map is more difficult since it is not clear how far an AV would stop in front of a stop line. In this case, the longitudinal error distance can only be determined as a combined error distance of the longitudinal stop error and the localization error similar to the lateral error distances in relation to the lane-centerline. Therefore, for the current evaluation, the longitudinal error is only calculated for pre-defined stop locations on the driving path.

### 2.4. Evaluation Datasets

This section introduces the ground-truth and the localization datasets.

#### 2.4.1. Ground-Truth Datasets

**Figure 3:**
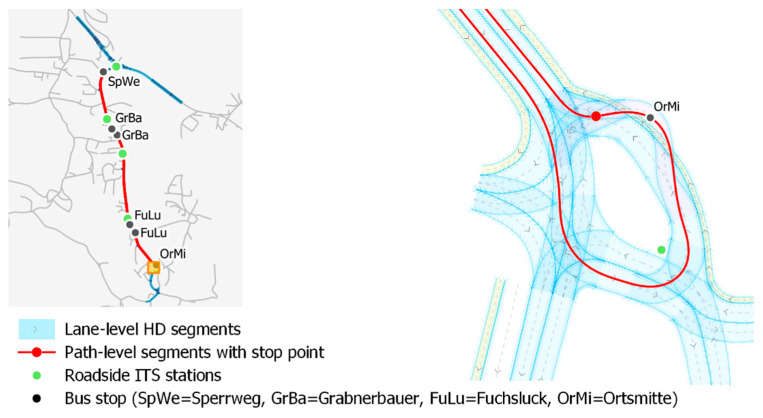
Overview of the test region including the different ground truths and a detailed view around Koppl Ortsmitte showing the driving path (red), the driving lanes of the Lanelet2 HD map (blue) and the generated lane-centerlines (grey dashed lines within lanes) as well as roadside ITS stations (green) and the bus stops (black).

As described earlier, for the evaluation of localization accuracy, two different ground-truth datasets are used. Both datasets represent a 1.4 km-long section of the municipal road L226 in the municipality of Koppl near Salzburg in Austria which is used for automated driving tests. An overview of the whole test track (red line) together with a detailed view of the driving path (red) and the HD map (light blue) at bus stop ‘Ortsmitte’ is visualized in Figure 3.

The static driving path of the EasyMile EZ10-Gen3 vehicle is used for the path-level ground truth (red line in Figure 3). During the deployment of an EasyMile EZ 10 shuttle, the driving path is acquired by a manual drive and manually edited afterwards so that the vehicle moves perfectly on the lane. This driving path is then used by the ADS of the EZ10-Gen3 for its path-following functionality. Therefore, it has to be accurate since the vehicle has to stay on the lane at any time. For this work, the driving path has been converted into a road graph structure with five-meter-long segments (the segment length has been chosen arbitrarily, other lengths are possible as well).

As lane-level ground truth dataset, an HD map in the Lanelet2 format has been used [25]. The HD map primarily represents the test track on the rural road L226 in Koppl (along the red line in Figure 3). For building the HD map, the road infrastructure has been mapped with an HD mapping vehicle. Afterwards, road features such as lane boundaries and lane markings have been extracted from the LiDAR point cloud with the software tool TopoDOT^®^ (https://new.certainty3d.com/, accessed on 5 July 2021). The accuracy of the extracted features has been validated by surveying 345 reference points manually. The validation revealed a mean accuracy of 0.07 m (sd: 0.1 m) for all validated features which satisfies the ground truth assumption. In order to be used for localization accuracy evaluation, a lane-accurate Lanelet2 HD map has been composed from the extracted geographic features using the JOSM editor (https://josm.openstreetmap.de/, accessed on 5 July 2021). Additionally, regulatory elements have been added so that a lane-level road graph could be derived. If lane markings were missing in reality, a virtual road middle axis (calculated from the right and left road boundaries) acts as left or right lane boundary. The lane-centerlines have been generated automatically as middle axis between the left and the right lane boundaries using the HD feature of the open-source tool Graphium (https://github.com/graphium-project/graphium/tree/feature-graphium_hd, accessed on 5 July 2021) for managing road graphs. Graphium has been extended to manage Lanelet2 HD maps.

For evaluating the C-ITS data from manually driven connected vehicles, the lane-level HD map has been extended by the intersection connecting the L226 municipal road to the B158 main road as well as 900 m of the road B158 (blue line in Figure 3). This was necessary since most trajectories from C-ITS-enabled vehicles have been recorded on this road section.

#### 2.4.2. Localization Datasets

In order to evaluate the methods, two different test datasets have been recorded. The first test dataset, called the “AV dataset”, has been generated by using the localization system of the EasyMile EZ10-Gen3 automated shuttle. The EZ10-Gen3 has the ability to follow a static driving path (ground truth) at a speed of 15 km/h at maximum. Localization accuracy of the EZ10-Gen3 vehicle may be measured by setting the localization data in relation to the static driving path. It has to be noted that for evaluating localization accuracy, the EZ10-Gen3 vehicle has to be operated in autonomous mode (since only in this mode it will follow the pre-defined driving path). Localization measurements during manually operated sections have been excluded from the accuracy evaluation. In addition, if the vehicle is not located exactly on the driving path after manual driving, after being set into autonomous mode again, the vehicle starts to get on its driving path. Therefore, the first 10 m after switching to autonomous mode have been excluded from the evaluation as well.

The localization system of the EZ10-Gen3 fuses data from four different sensor systems. A GNSS-RTK receiver, located on the vehicle’s geometrical center on the roof top (reference point) along with one LiDAR sensor on the front roof top that positions the shuttle by using SLAM technology. The GNSS signal has been improved using the HxGN SmartNet RTK service (https://hxgnsmartnet.com/, accessed on 5 July 2021) to correct ionospheric and tropospheric distortions as well as satellite clock bias and orbital errors [28]. The next base station is 3.5 km away. Further, the EZ10-Gen3 features an Inertial Measurement Unit (IMU) and an odometer to improve localization performance. Due to commercial interests of EasyMile, the exact fusion algorithm is not revealed. However, the fused localization measurements are made available via an API at 2 Hz frequency.

**Figure 4:**
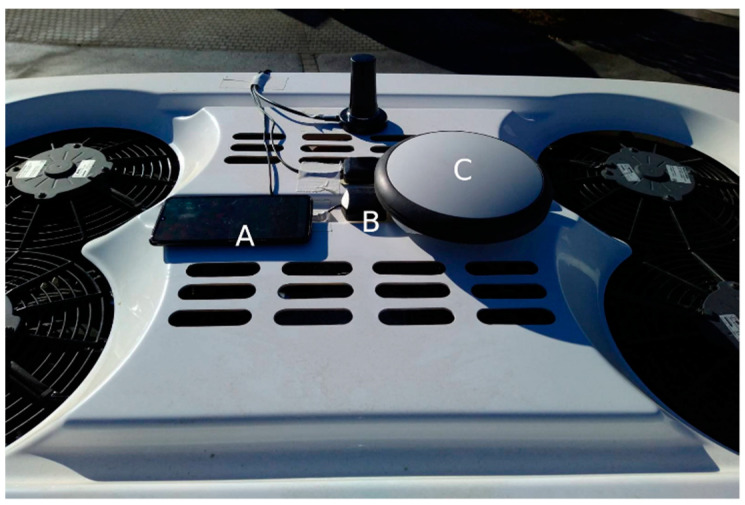
Mounted sensor setup on the roof top of the EZ10-Gen3 shuttle: (**A**) Xiaomi Mi 9 Smartphone, (**B**) GNSS sensor of the C-ITS-OBU and (**C**) Leica Zeno GG04 plus antenna. The front of the vehicle is to the right of the picture.

In order to set the EZ10-Gen3 results in contrast to results of other localization systems, three additional localization systems have been mounted on the EZ10-Gen3 vehicle (Figure 4). The systems have been placed almost to the reference point on the roof top of the EZ10-Gen3 shuttle: (a) the Leica Geosystems Zeno GG04 Smart Antenna (https://leica-geosystems.com/en-us/products/gis-collectors/smart-antennas/leica-zeno-gg04-plus, accessed on 5 July 2021) (geodetic grade Multi-GNSS/RTK receiver) (Heerbrugg 9435, Switzerland), (b) Xiaomi Mi 9 Smartphone (https://www.mi.com/global/mi9/, accessed on 5 July 2021) (consumer grade IMU-supported L1/L5 multi GNSS receiver) (Beijing 100085, China), and (c) a prototypical C-ITS on-board unit (OBU) in development from Kapsch TrafficCom (Vienna 1120, Austria) (EVK-3300 V2X Evaluation Kit) using two different consumer grade Multi-GNSS-receivers (ublox NEO-M8L and ublox C94-M8P-3 with NEO-M8P-2 chipset). The Leica Zeno GG04 plus GNSS/RTK antenna records locations with the aid of a RTK signal to reach centimeter accuracy. It can read the satellite signals and frequencies of GPS (L1, L2, L2C, L5), Glonass (L1, L2), BeiDou (B1, B2, B31), Galileo (E1, E5a, E5b, Alt-BOC, E61), QZSS2 and SBAS (WAAS, EGNOS, MSAS, GAGAN) [29]. For the evaluation of the longitudinal accuracy, an offset of 0.09 m longitudinal offset has been considered to compensate the distance between the device antenna’s phase center and the reference point. The Xiaomi Mi9 is one of the few smartphones featuring multiple GNSS frequency bands to improve localization quality at the time of the evaluation. The Xiaomi Mi9 can read GPS (L1 + L5), Galileo (E1 + E5a), GLONASS (L1) and Beidou (B1) signals and improves the localization with IMU data. The offset of the device antenna’s phase center to the vehicle’s reference point (0.08 m longitudinal and 0.055 m lateral offset) can be neglected due to the overall lower accuracy. The location data recorded by the C-ITS OBU has been acquired using two different ways: (1) using the internal consumer grade GNSS receiver (ublox NEO-M8L), and (2) using a consumer grade external GNSS receiver (ublox C94-M8P-3 with NEO-M8P-2 chipset) being augmented by GNSS correction data received from a local C-ITS-based GNSS Positioning Correction (GPC) Augmentation Service [30]. GNSS corrections have been calculated by using the roadside ITS stations as GNSS reference stations (RIS-9160 roadside ITS station from Kapsch TrafficCom with a prototypical implementation of a RTK-enabled GNSS reference station using an ublox NEO-M8P-2 chipset) and have been transmitted to the OBU using ETSI ITS-G5 RTCMEM [31]. It has to be noted that for RTK-supported localization systems, measurements with missing RTK correction signals have been excluded (this applies to the Leica GNSS antenna and the C-ITS OBU).

For generating the test dataset for the EZ10-Gen3 localization system as well as the additional localization systems mounted on the EZ10-Gen3 vehicle, a three-day evaluation period from 30 November to 2 December, 2020 has been chosen. Data has been recorded during three-hour-sessions with two one-hour breaks in-between in order to mind different satellite constellations as recommended by other studies [32]. Table 2 gives an overview of the EZ10-Gen3 data and the three mounted localization systems. Position data with missing RTK correction and data recorded in manual driving mode is excluded.

To demonstrate the wider applicability of the approach, a second test dataset of manually driven SAE level 0 vehicles, called the “C-ITS dataset”, has been used. The localization measurements of the C-ITS dataset have been collected from C-ITS-enabled vehicles sending ETSI ITS-G5 CAM messages [20]. Starting in September 2020, five roadside ITS stations (R-ITS-S, localized in Figure 3) are collecting CAM messages from C-ITS-enabled vehicles passing the R-ITS-S. Vehicles such as the Volkswagen Golf 8, ID.3 or ID.4 are already equipped with C-ITS-enabled onboard units and are sending continuously CAM messages (if the Car2X functionality has not been deactivated by the user). During the test period from week 38, 2020 to week 11, 2021, 664 trajectories from C-ITS enabled vehicles have been recorded. It has to be noted that vehicles periodically change their identification for privacy reasons. 127 trajectories originating from the C-ITS OBU installed in the EZ10-Gen3 are already included in the “AV dataset” and have therefore been excluded from the “C-ITS dataset”. The remaining 537 trajectories cover a distance of 154.9 km and a duration of 154 min.

## 3. Results

**Figure 5:**
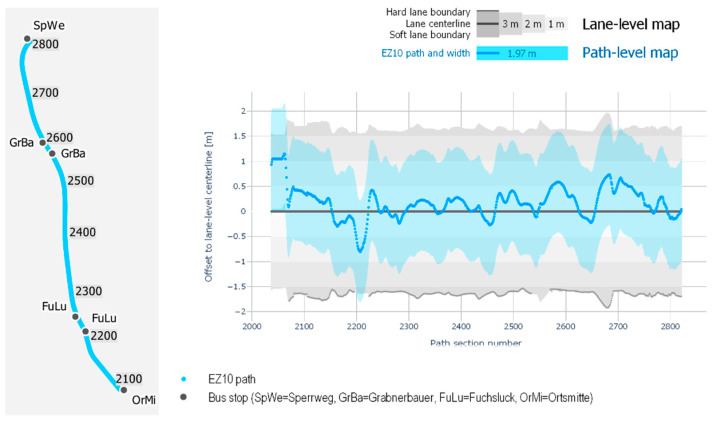
Comparison of the driving path (blue line), lane-centerline (solid black line), left (positive values) and right (negative values) lane boundaries (solid grey boundaries) as well as the error thresholds in relationship to the HD map for the test track.

In order to evaluate the proposed methods for estimating localization accuracy, we have conducted several experiments comparing the localization measurements with the ground-truth datasets. Before presenting the evaluation results, we first evaluate the ground-truth datasets by compare the driving path to the lane-accurate HD map. While Figure 1 provides an abstract view of the widths and distance metrics of a driving lane, Figure 5 shows the exact widths of the driving lane modelled in the ground-truth dataset. The figure reveals that the driving path (blue horizontal line) is mostly within a 0.5 m corridor along the lane-centerline (black horizontal line in the middle). The figure also reveals that the driving path tends towards the road-centerline as the offset tends to the left of the lane centerline (upper half of the chart). The vehicle width (1.97 m, blue area) is shown as corridor along the driving path and shows whether it keeps within the lane corridor which is indicated with 1 m, 2 m, and 3 m wide corridors (grey colors with increasing lightness) along the lane-centerline. Additionally, the solid dark grey line at the bottom indicates hard road boundaries that can or may not be crossed. Hard road boundaries include curbstones, the road border (e.g., grass) and solid lane markings. They mainly occur at the right boundary where the road lane is next to the walkway or banquet. Concerning localization accuracy, the error distance including the vehicle’s width and the vehicle’s deviation from the lane-centerline may never overlap the hard lane boundaries.

### 3.1. Evaluating Localization Accuracies in Relation to the Driving-Path

The first evaluation sets the position trajectories of the “AV dataset” in relation to the static driving path of the EZ10-Gen3. Table 3 gives an overview of the mean of lateral Euclidian error distances of the localization measurements, the standard deviation (sd), and different confidence levels (p50, p95, p99, p99.9) in relation to the driving path per localization system. The table reveals that only the localization system of the EZ10-Gen3 is capable to meet the localization requirements for AV’s proposed in [3] (0.1 m; 95% confidence). As expected, the localization system of the EZ10-Gen3 achieves the lowest median of error distances of 0.013 m. The error distances for the p95 and p99-percentiles stay still below 0.1 m error threshold. Since the autonomous driving functionality of the EZ10-Gen3 is implemented to stay within a 0.3 m corridor of the driving path (alert limit), the low error distance of the EZ10-Gen3 can be expected. If the vehicle detects a lateral localization error of more than 0.1 m from the driving path, it slows down. If the lateral error distance exceeds 0.3 m (alert limit), the vehicle stops until a better localization is available or until the operator moves the vehicle to a location with a better localization manually. This behavior is in full accordance with the localization requirements for AVs on local roads proposed by [3]. Offsets may occur due to incorrect GNSS signals and/or LiDAR localization or if the vehicle drifts off the path while driving on gravel or similar surfaces, especially at turns with low radius. This situation occurs regularly near the station Sperrweg in the North of the test track.

Beside the EZ10 Gen3, also the position measurements of the Leica GG04plus stay below the 0.1 m threshold for the p50-percentile, but exceed the threshold for the p95-percentile. Concerning the Leica Zeno GG04 plus it has to be noted that this localization system only uses Multi-GNSS/RTK-localization without INS-support or any other sensor fusion. All other tested localization systems, although achieving reasonable localization results, do not meet the accuracy requirements of an AV. The results also reveal that the GNSS corrections obviously improve the accuracy of the C-ITS OBU (it has to be noted that only C-ITS OBU measurements with the states RTK-float (carrier phase floating point solution) and RTK-fixed (carrier phase integer ambiguity resolution) have been considered for the evaluation). However, a higher accuracy will be difficult to achieve with a low-cost GNSS chipset. Furthermore, the applied C-ITS OBU is still under development and better results are expected in the future.

In contrast to the measurement-based evaluation, the distance-based evaluation weights the lateral error distances by the driving distance. This weighting lowers localization errors during slow drives or stops (e.g., visible as decreased averages and standard deviation). Table 4 shows the comparison of the absolute error distances to the driving path. While the p50- and p95-percentiles for the EZ10-Gen3 are nearly identical, the value for the p99.9-percentile is significantly lower and still stays below the 0.1 m error threshold. The Leica Zeno GG04 plus results stay below the 0.1 m threshold only for the p50-percentile. The p95- and p99 percentiles stay below a 0.3 m threshold. Overall, deviations most likely occur in case of stops if the localization system produces measurements at a fixed sampling interval. If localization systems suppress localization measurements in case of stops, deviations between the two methods are minimal. Nevertheless, especially at lower speeds, results can significantly deviate in comparison to a measurement-based evaluation.

**Figure 6:**
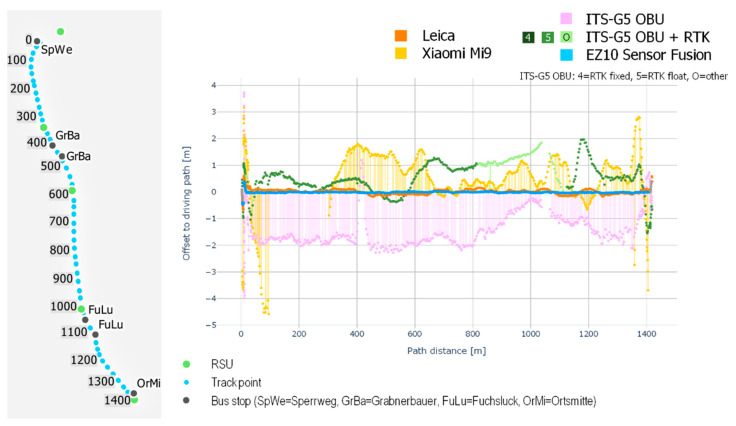
Lateral error distances (in meters) in relation to the driving-path for the different localization systems; data of an exemplary EZ10-Gen3 test drive in Koppl from Sperrweg (North) to Ortsmitte (South).

For analyzing lateral localization accuracies along the test track, a visualization of distance errors along the driving path has been chosen. Figure 6 shows the lateral distance errors of the four different localization systems (y-axis) for an exemplary test drive along the test track in Koppl from the bus stop Sperrweg (North) to the bus stop Ortsmitte (South) in relation to the driving-path (in the figure also C-ITS measurements without RTK correction are included). The figure shows that the lateral distance errors of the EZ10-Gen3 and the Leica Zeno GG04 plus antenna stay near the zero value on the y-axis for the whole track while all other localization systems show variable error rates. The Xiaomi Mi9 shows distance errors up to the 5 m threshold while the localization system of the C-ITS OBU stays within an error distance of 2.5 m.

### 3.2. Evaluating Localization Accuracies in Relation to the Lane-Centerline

If the exact driving-path is not known or not available for the localization accuracy evaluation, a lane-level evaluation can be applied. As described in Section 2.3, this method sets the localization measurements in relation to the lane-centerline of a ground truth HD map. Again, measurement-based as well as the distance-based evaluations are compared.

Table 5 gives an overview of the lane-level lateral error distances for the five different localization systems. As has already been demonstrated in Figure 5, the driving path of the EZ10-Gen3 is not exactly following the lane-centerline. Therefore, the lateral error distances in Table 5 are a combination of the driving path’s deviation from the centerline and the localization error. If we consider the vehicle width of the EZ10 Gen3 of 1.97 m and assume an average lane width of 3.5 m, the combined distance error of the AV may not exceed 0.765 m from the lane-centerline so that the vehicle is able to stay within the lane boundaries. Looking at the results in Table 5, for the EZ10-Gen3 and Leica GG04plus localization systems this is valid for the p50- and the p95-percentiles but not for higher confidence levels. As gets visible from Figure 5, the driving path is neither aligned to the lane-centerline nor is the lane width always 3.5 m. Therefore, it is possible that the combined error distance exceeds the defined error threshold, even if the localization error is below the error threshold. This result clearly indicates the supremacy of the path-related evaluation or the calculation of actual lane boundary overlaps as presented in Section 3.3.

While all other localization systems are not able to cope with the lane-level accuracy requirements, the localization system of the C-ITS +RTK OBU exceeds the error threshold for the p50-percentile by 0.021 m only.

Again, beside the measurement-based error distances, the distance-based lateral error distances have been calculated (Table 6). The results confirm the driving path-related results. The distance errors of the different percentiles are a bit lower compared to the measurement-based ones since erroneous measurements during slowly driven route parts or stops are weighted lower (as indicated by lower mean errors and standard deviations). The EZ10-Gen3 and the Leica GG04plus localization systems stay within the error threshold of 0.765 m at the 95% confidence level. Interestingly, up to the p99-percentile, the Leica GG04plus shows similar results to the EZ10-Gen3 with a slightly better result compared to the EZ10-Gen3 for the p99-percentile. For the other localization systems, the overall picture from the measurement-based evaluation is confirmed.

**Figure 7:**
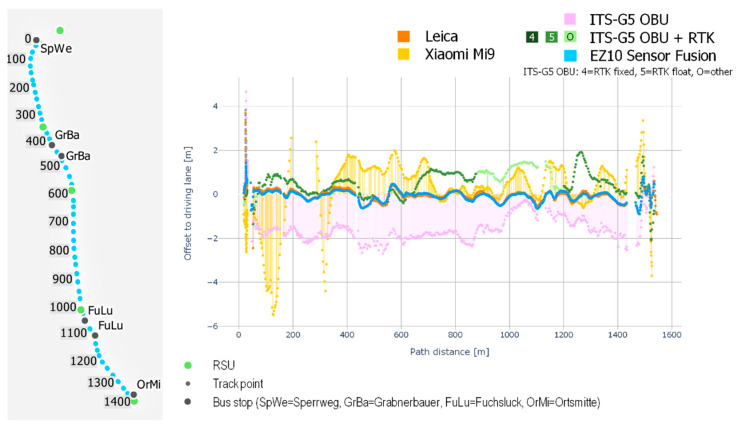
Lateral error distances (in meters) to the lane-centerline for the different localization systems; data of an exemplary EZ10-Gen3 test drive in Koppl from Sperrweg (North) to Ortsmitte (South).

Figure 7 shows the lateral error distances of the five localization systems in relation to the lane-centerline along the test track for one exemplary test drive. As expected, since the driving-path of the EZ10-Gen3 deviates from the lane-centerline, also the combined distance error deviates (blue line), but still stays below the 0.765 m error threshold. Also, the Leica Zeno GG04 plus shows a similar result while the three other localization systems reveal significantly higher error rates.

### 3.3. Evaluating Localization Accuracies by Lane Boundary Overlaps

Besides calculating localization accuracies in relation to the driving-path and the lane-centerline, the third evaluated method calculates the longitudinal absolute distance and ratio of left and right lane-boundary overlaps of the AV’s geographically oriented geometrical body. In the Lanelet2 HD map, the lane boundaries are modelled either as hard or soft lane boundaries. Hard lane boundaries should not be crossed (e.g., type curbstone, road border or solid road marking). The assumption is that vehicles should never cross such hard boundaries during driving. On the other hand, soft lane boundaries such as dotted road markings may be crossed, but only in specific cases. Therefore, during a usual drive (such as the test drives), the vehicle should stay within both types of boundaries and the overlap of the localized oriented vehicle body should not overlap the boundaries. Table 7 shows the absolute and relative driving distance with left and right boundary overlaps per localization system. As the test track in Koppl primarily follows a two-lane rural road (one lane per direction) and there is no solid lane marking in-between, there are almost no overlaps with hard boundaries to the left (towards the road middle axis). In contrast, the right lane boundary usually represents the road border, a curbstone or a solid road marking. The presented distances are based on the longitudinal distances of the lane-centerline. Since hard lane boundaries should not be crossed, hard lane boundary overlaps in Table 7 most probably are a result of localization errors. While the EZ10-Gen3 trajectories reveal no hard lane boundary overlaps, the other devices have an overlap ratio above 10%. As expected, hard lane boundary overlaps mainly occur to the right whereas overlaps to the left are mainly soft boundary overlaps. Another notable observation are the varying overlap error distances to the left (towards the road middle axis) and right (towards the road border) of the Xiaomi Mi9, C-ITS and C-ITS RTK localization systems. Since the data has been acquired at the same time and the Xiaomi Mi9 and the C-ITS systems show similar deviations to the right, the reason for the left deviation of the C-ITS RTK system can only be found in the RTK correction. Since these deviations appear during the whole test period and along the whole test track (with an emphasis on the Northern parts) inappropriate satellite constellations are only hardly be accountable and no conclusive explanation has been identified.

### 3.4. Evaluating Longitudinal Localization Accuracies

Along the EZ10 driving path for the Koppl test track, there are two stop locations that can be used as ground-truth for the evaluation of longitudinal localization accuracies. The first stop is located after the station ‘Ortsmitte’ before entering the road L226 (indicated in Figure 3). The second stop is located near the station ‘Sperrweg’ before turning into the same road. These stop locations are exactly located on the driving path of the EZ10-Gen3. While the first is a mandatory stop, the second one represents a yield where the vehicle is allowed to resume without stopping. From the “AV dataset”, 11 (first stop) and 7 (second stop) trajectories contain stops in autonomous mode and therefore can be used for the evaluation. All other trajectories have been discarded due to manual driving before or at the stop location.

Table 8 shows the linearly referenced stop locations (covered distance on path before stop) as well as the longitudinal error distances on the driving path. The linear references of stop locations refer to the driven distance on the path before the stop. Both stop locations are shortly after the start of the path (11.29 and 24.22 m). The results indicate a median longitudinal Euclidean error distance of 0.06 m (first stop) and 0.04 m (second stop) for the EZ10-Gen3. The trajectories recorded with the Leica GG04plus antenna reveal a longitudinal offset between 0.12 and 0.20 m. The values of this device have been corrected by 0.09 m to compensate the longitudinal distance between the antenna and the vehicle’s longitudinal middle axis. The other localization systems reveal a longitudinal error distance above 0.5 m.

### 3.5. Evaluating Localization Accuracies of Manual Drives

In order to show the wider applicability of the approach, the accuracy evaluation has been applied to the “C-ITS dataset” containing position trajectories from manually driven, connected vehicles. As described earlier, the second trajectory dataset has been generated from ETSI ITS-G5 CAM messages received from C-ITS-enabled vehicles via five roadside ITS stations. Due to the CAM origin of the localization data, the actual vehicles and their localization systems are not known. However, since the availability of localization data from connected vehicles continuously increases and for many C-ITS use cases such as Intersection Collision Risk Warning (ICRW) [22] location accuracy matters, applying the proposed localization accuracy evaluation to this kind of data is considered highly relevant as well. Moreover, in comparison to the “AV dataset”, this dataset also contains data from vehicles moving at higher speeds up to 80 km/h.

**Figure 8:**
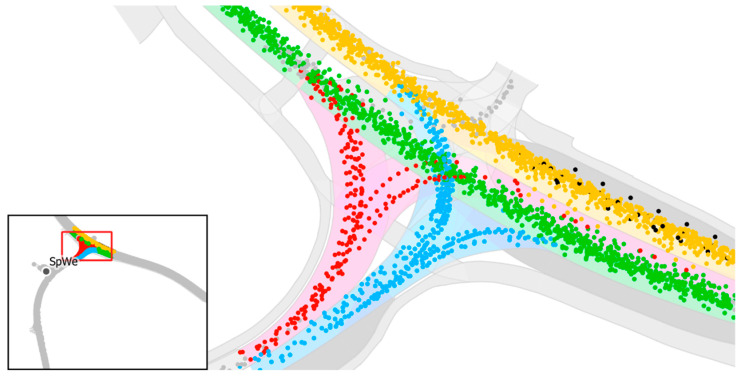
Section of the B158 road showing the lane-level HD map including driving lanes and bus stops and the localization measurements received via ETSI ITS-G5 CAM messages. The colors link track points to their map-matched HD segments.

Figure 8 shows the lane-level HD map of the intersection connecting the autonomous driving test track on the L226 municipal road with the B158 major road and the map-matched localization measurements of the “C-ITS dataset” on this intersection. The lane-level HD map excerpt contains one driving lane on the B158 in each direction (green and yellow), lanes for safe turning to and from the L226 (red and blue) as well as separate bus lanes (dark grey) leading to the bus stops on each side of the road. The light grey areas represent pedestrian and bicycle paths.

Since the actual driving-lane is not known, each measurement is matched to the lane-centerline with the lowest normal Euclidean distance in case that the regulatory elements allow such a driving maneuver.

The lane-level accuracy evaluation of position measurements reveals a median Euclidean distance error of 1.157 m in relation to the matched lane-centerline (Table 9). As expected, the distance-based evaluation reveals a slightly better result with a median distance error of 1.038 m. Overall, the results show that the error threshold for lane accuracy (0.75 m; considering a lane width of 3.5 m and a vehicle width of 2 m) is by far not achieved.

In addition to the lateral distance error analysis, a lane-boundary overlap analysis has been performed for the C-ITS test dataset as well (Table 10). Since the driving lanes at this part of the road are slightly curved (Figure 8), the localization measurements have a tendency towards the North-East as the vehicles probably move on the inner side of the slight curve. Since there are additional lanes on both sides of the main driving lane, the lane boundaries are only soft boundaries and it is possible for the vehicles to move near the lane boundaries. The results of the lane-boundary overlap analysis in Table 10 show this tendency with higher overlaps of soft lane boundaries. Again, the results show that the lane accuracy requirements (at least no hard lane boundary overlaps) are not met.

## 4. Discussion

The evaluation results demonstrate the overall applicability of the proposed methods for evaluating lateral and longitudinal localization accuracy of AVs (and also connected vehicles). The evaluation of position measurements in relation to the exact driving-path is considered the ‘gold standard’ for evaluating localization accuracy. In the case of the EZ10-Gen3, this kind of evaluation is possible since the driving-path is static and the vehicle is programmed to follow the driving-path at best effort. Additionally, if the driving path contains programmed stop points, these can be used to evaluate longitudinal localization accuracy. If the driving-path is dynamically calculated by the vehicle, a lane-level evaluation is considered more appropriate. Concerning the lane-level evaluation, both proposed methods, the lane-centerline method as well as the lane-boundary overlap method, are appropriate methods to estimate localization accuracy with respect to a HD map ground truth. The prerequisite for this kind of evaluation is a lane-accurate HD map with an absolute geographical accuracy below 0.1 m (0.01 m would be best as the ground truth should always be a decade smaller than the accuracy threshold [3], but is hard to achieve and validate). The lane-centerline method uses the centerline as ‘ideal’ driving path and estimates a combined error distance of the driving and the localization-induced deviations from the centerline. This method is especially useful in order to determine whether the localization system is capable to localize the AV within the lane boundaries. However, in contrast to the driving-path evaluation, only the combined error distance (deviation from the centerline and localization error) can be evaluated. The localization error cannot be evaluated separately. Therefore, if the driving path is not known, the lane-boundary overlap method is more appropriate since it evaluates the exact overlaps of the localized oriented vehicle body in relation to hard or soft lane boundaries. If the types of lane boundaries are available, the last method allows for a semantically richer accuracy evaluation. Furthermore, if the localization system is capable of high accuracy below 0.1 m, the evaluation of hard lane-boundary overlaps can also be used to evaluate whether an AV adheres to driving rules.

Concerning the differences between the measurement- or distance-based evaluation methods, results do not show a clear advantage for one of the two methods. It depends what actually should be measured. In case of highly-precise localization systems, increasing distance errors at low speeds or vehicle stops will typically not occur. However, the results of the C-ITS dataset indicate that especially for higher speeds, the weighting of lateral distance errors with the longitudinal driving distance can lead to lower overall distance errors at different confidence levels. Since key performance indicators for AVs such as disengagements are also measured distance-based, the distance-based evaluation of localization accuracy seems more appropriate. Related works also propose to use vehicles’ operation hours as reference for calculating localization integrity [3]. While easily to be calculated, this metric only makes sense for large datasets with millions of driving hours.

Concerning the results of the different localization systems, only the EZ10-Gen3 and the Leica Zeno GG04 plus systems are showing results coping with the requirements of AVs proposed by [3], at least at rather low driving speeds up to 15 km/h. Only these highly-accurate localization systems achieve accuracies below 0.1 m at a confidence level of 95%. However, since the accuracy of localization systems has been tested in an intermediate environment (between open and urban) and not in an urban environment, it gets clear how challenging the localization requirements for an AV are. While an accuracy of 0.1 m at 95% confidence seems feasible under good conditions, achieving these confidence levels under all possible situations and environments challenges the localization systems, even if they are fusing data from several sensor systems such as the EZ10-Gen3. However, since similar localization accuracies as for AVs are also needed for C-ITS use cases of manually-driven vehicles such as Intersection Collision Risk Warning (ICRW), it makes sense to apply the proposed method to localization data of manually driven connected vehicles as well. Results show that the achieved localization accuracies of the current C-ITS-equipped vehicles are not coping with the localization accuracy requirements of AVs. Even lane-level accuracies below 0.75 m at 95% confidence are out of scope for current vehicles on the road. One promising approach is to augment the position measurements with GNSS correction data provided via roadside ITS stations. Given the used consumer grade GNSS receiver in the OBU, the achieved results indicate the potentials of this approach for the future. Using geodetic grade GNSS receivers can further improve localization accuracy as indicated by a related study [33]. Since C-ITS-originated data will be broadly available via ETSI ITS-G5 CAM messages in the future, the study can be repeated at any time in order to evaluate localization accuracies of future C-ITS-enabled vehicles.

## 5. Conclusions

Connected and automated vehicles are in need of accurate and reliable localization. While numerous localization techniques have been proposed during the last decade, the question of how to validate accuracy and reliability of these techniques in real-world environments remains unaddressed. The current work closes this gap by not focusing on new localization techniques, but by proposing and evaluating methods which can be used for validating localization accuracy of AVs in relation to ground truth datasets. Therefore, the main contribution of this work is on the accuracy evaluation methodologies, while two trajectory test datasets demonstrate the broad applicability of the approach. Furthermore, the proposed evaluation methods complement the work by [3] allowing to evaluate the proposed accuracy and confidence requirements of automated vehicles (it also complements the work by [12] proposing algorithms to calculate integrity levels). Therefore, together with [3] and [12], the current work can act as a welcome foundation for localization performance validation for AVs and connected vehicles.

Beside the aforementioned contributions, the proposed approach also has some limitations. One of the main challenges arises from treating the localization system of an AV as black box. While the approach to relate the location measurements to a static driving path allows accuracy evaluations for arbitrary position trajectories, the ambiguity of the relation between the localization error and the path-following capability (actuation error) of the AV remains. Another challenge arises from the chart or map error. Since also a HD map typically contains a certain location error, it may only hardly be considered as ground truth and for the accuracy evaluation the map error has to be considered as well. However, since the error varies between different sections and elements of the HD map, it can hardly be determined. Also, achieving HD map accuracies at 0.01 m (which is needed for validating localization accuracies of 0.1 m) is an outstanding challenge, even for professional mapping agencies. A third challenge arises from the test environment and the amount of test data being used for the accuracy evaluation. In this study, only one test track in a rural area and a limited number of test drives has been used. In order to gain broader results, a larger amount of test drives in more complex urban and non-urban environments has to be conducted.

Concerning future work, it would be worthwhile to evaluate additional parameters such as vertical error distances or the accuracy of vehicle orientations. Moreover, the study should be repeated with different autonomous vehicles on different test tracks in more challenging environments. Generally, due to the continuous progress in localization technologies, it makes sense to regularly repeat the study including future localization systems or data from partly or highly automated and connected vehicles. Moreover, in future studies, it would be worth to apply the method to other connected traffic participants such as bicyclists.

## Figures and Tables

**Table 1:** Possible accuracy evaluations with respect to SAE J3016^TM^ automation levels.

	SAE 0	SAE 1	SAE 2	SAE 3	SAE 4	SAE 5
Path accuracy				X	X	X
Lane accuracy	X	X	X	X	X	X
Longitudinal accuracy				X	X	X

**Table 2:** Overview of the recorded test datasets.

Device	Distance [km]	Duration [min]	Sampling Interval Median [s]
EZ10-Gen3	73.9	400.2	0.5
Leica GG04plus	28.7	161.6	1.0
Xiaomi Mi9	62.9	340.6	1.0
C-ITS OBU RTK	42.1	210.0	1.0
C-ITS OBU	66.3	359.1	1.0

**Table 3:** Measurement-based lateral error distances in relation to the driving-path for the first localization dataset generated by test drives of the EZ10-Gen3 and mounted additional localization systems; distances in meters.

Device	TP Count	Mean	sd	p50	p95	p99	p99.9
EZ10-Gen3	48,070	0.017	0.025	0.013	0.044	0.071	0.349
Leica GG04plus	9699	0.093	0.120	0.079	0.228	0.319	0.929
Xiaomi Mi9	20,440	1.318	1.417	1.175	2.970	3.526	4.615
C-ITS + RTK	12,502	0.851	0.944	0.724	2.093	2.735	3.894
C-ITS	21,417	1.652	1.540	1.487	3.790	4.620	5.255

**Table 4:** Distance-weighted lateral error distances in relation to the driving-path for the first localization dataset generated by test drives of the EZ10-Gen3 and mounted additional localization systems (distances in meters).

Device	Distance	Mean	sd	p50	p95	p99	p99.9
EZ10-Gen3	73,872	0.016	0.014	0.013	0.042	0.061	0.086
Leica GG04plus	28,711	0.084	0.062	0.073	0.196	0.259	0.458
Xiaomi Mi9	62,831	1.243	0.875	1.092	2.830	3.390	4.260
C-ITS + RTK	42,088	0.849	0.644	0.727	2.051	2.724	3.893
C-ITS	66,307	1.629	1.083	1.487	3.665	4.394	4.851

**Table 5:** Measurement-based lateral error distances for the first localization dataset in relation to the lane-centerline generated by test drives of the EZ10-Gen3 and mounted additional localization systems; distances in meters.

Device	TP Count	Mean	sd	p50	p95	p99	p99.9
EZ10-Gen3	45,064	0.223	0.299	0.170	0.611	0.949	1.088
Leica GG04plus	9066	0.251	0.341	0.191	0.641	1.193	1.418
Xiaomi Mi9	19,386	1.282	1.369	1.105	2.959	3.671	4.742
C-ITS + RTK	12,259	0.887	0.987	0.786	2.133	2.897	3.960
C-ITS	21,196	1.723	1.580	1.550	3.913	4.646	6.334

**Table 6:** Distance-weighted lateral error distances in relation to the lane-centerline for the first localization dataset generated by test drives of the EZ10-Gen3 and mounted additional localization systems; distances in meters.

Device	Distance	Mean	sd	p50	p95	p99	p99.9
EZ10-Gen3	73,872	0.209	0.175	0.168	0.565	0.770	1.030
Leica GG04plus	28,711	0.235	0.195	0.189	0.579	0.727	1.579
Xiaomi Mi9	62,831	1.237	0.884	1.066	2.821	3.528	4.666
C-ITS + RTK	42,088	0.872	0.640	0.787	2.040	2.717	3.932
C-ITS	70,149	1.739	1.183	1.580	3.904	4.675	6.511

**Table 7:** Lane-boundary overlap distances and ratios between the vehicle geometrical body and hard or soft lane boundaries; data of all EZ10-Gen3 drives in Koppl between Nov. 30 and Dec. 2, 2020 (distances in kilometers).

Device	Total Distance	Left Overlap Distance	Right Overlap Distance
**Hard lane boundaries only**			
EZ10-Gen3	73.568	0 (0.00%)	0.000 (00.00%)
Leica GG04plus	56.633	0 (0.00%)	6.170 (10.92%)
Xiaomi Mi9	63.029	0 (0.00%)	26.759 (42.56%)
C-ITS RTK	69.586	0 (0.00%)	7.634 (10.97%)
C-ITS	69.429	0.443 (0.64%)	30.252 (43.57%)
**Soft lane boundaries only**			
EZ10-Gen3	73.568	0.281 (00.38%)	1.329 (01.81%)
Leica GG04plus	56.633	6.481 (11.47%)	1.297 (02.30%)
Xiaomi Mi9	63.029	8.315 (13.22%)	2.889 (04.59%)
C-ITS RTK	69.586	29.713 (42.70%)	1.577 (02.27%)
C-ITS	69.429	5.732 (08.26%)	5.037 (07.26%)
**All lane boundary types**			
EZ10-Gen3	73.568	0.281 (00.38%)	1.329 (01.81%)
Leica GG04plus	56.633	6.481 (11.47%)	7.466 (13.22%)
Xiaomi Mi9	63.029	8.315 (13.22%)	29.648 (47.15%)
C-ITS RTK	69.586	29.713 (42.70%)	9.211 (13.24%)
C-ITS	69.429	6.175 (08.89%)	35.289 (50.83%)

**Table 8:** Longitudinal median of error distances of test drives for the two stop locations along the driving path (distances in meters).

Device	Stop ‘Ortsmitte’	Stop ‘Sperrweg’
Ground truth	11.29	24.22
EZ10-Gen3	11.35 (+0.06)	24.18 (−0.04)
Leica GG04plus	11.41 (+0.12)	24.02 (−0.20)
Xiaomi Mi9	9.27 (−2.02)	23.26 (−0.96)
C-ITS OBU RTK	10.15 (−1.14)	23.68 (−0.54)
C-ITS OBU	9.91 (−1.38)	22.17 (−2.05)

**Table 9:** Comparison of the distance errors of the C-ITS test dataset in relation to the lane-centerline of the HD map (distances in meters).

Method	Count	Mean	sd	p50	p95	p99	p99.9
Measurement-based	22,252 TPs	1.303	1.603	1.157	3.014	4.611	5.234
Distance-based	106.961 km	1.184	0.863	1.038	2.610	4.204	4.950

**Table 10:** Overlap between localized and oriented vehicle body and lane boundaries for the C-ITS test dataset.

Type of Boundary	Total Matched Distance (km)	Left Overlap Distance (km) and Ratio	Right Overlap Distance (km) and Ratio
All lane boundaries	151.402	38.561 (25.47%)	51.860 (34.25%)
Hard lane boundaries only		8.983 (05.93%)	41.649 (27.51%)
Soft lane boundaries only		29.578 (19.54%)	10.210 (06.74%)

## Data Availability

In order to foster reproducibility, the used data for generating the evaluation results are provided online at: https://doi.org/10.17605/OSF.IO/DN2QH, accessed on 27 August 2021. The position trajectories are provided as JSON files. The driving path is provided as GPX and JSON file. The HD map is provided in Lanelet2 format.
